# Vibrational and electrical properties of Cu_2−x_Te films: experimental data and first principle calculations

**DOI:** 10.1038/s41598-018-26461-x

**Published:** 2018-05-25

**Authors:** J. U. Salmón-Gamboa, A. H. Barajas-Aguilar, L. I. Ruiz-Ortega, A. M. Garay-Tapia, S. J. Jiménez-Sandoval

**Affiliations:** 1 Department of Physics, King’s College London, Strand, London, WC2R 2LS UK; 2 Centro de Investigación y de Estudios Avanzados del I.P.N. (Cinvestav), Unidad Querétaro, Libramiento Norponiente No. 2000, Frac. Real de Juriquilla, Querétaro, Qro. C.P. 76230 Mexico; 3 Centro de Investigación en Materiales Avanzados (CIMAV), Unidad Monterrey, Alianza Norte 202, Parque PIIT, Apodaca, Nuevo León, C.P. 66600 Mexico

## Abstract

Vibrational and electrical properties of sputtered films of the copper telluride system are presented. Despite of its technological importance in photovoltaics, the fundamental properties of copper tellurides are poorly understood. Films were deposited at 200 °C by rf sputtering from targets containing mixtures of copper and tellurium powders at nominal concentrations of Cu_1.25_Te, Cu_1.5_Te, Cu_1.75_Te and Cu_2_Te. Remarkably for the copper telluride system, it was possible to obtain single-phase vulcanite (CuTe) from the Cu_1.25_Te target. Two-phase mixtures of rickardite (Cu_7_Te_5_) and weissite (Cu_2−x_Te) were achieved for other cases. Raman spectra were obtained using two laser lines: 633 and 488 nm. Density functional theory was employed to calculate the phonon dispersion curves and density of states for vulcanite. The Raman bands were in good correspondence with the calculated frequencies. In general, the Raman spectra consisted of high-intensity totally symmetric modes superimposed on monotonically decaying signals. These were explained in terms of three contributing phenomena: convolution of vibrational normal modes, phonon-coupled charge density fluctuations and time-varying local-field contributions to the electric susceptibility. Studies on the conductivity, mobility and carrier concentration were carried out by the Van der Pauw method. Micro/nano scale surface potential studies were performed through Kelvin probe force microscopy mapping.

## Introduction

Anticipating the depletion of fossil fuels, the efforts directed to harvest solar energy by solar cells have been increasing during the past few decades. Particularly, thin film based solar cells have shown promising results towards higher efficiencies and cost reduction, compared to the conventional first generation solar cells based on single-crystal Si wafers^1^. Recently, Cu_2−x_Te thin films have shown interesting properties regarding their usage as back contacts for CdTe thin film solar cells^2–6^. Nevertheless, despite of the many studies on thin films composed by the Cu_2−x_Te system fabricated by various methods, a comprehensive study is yet missing, principally on the technologically important sputtered films. Minor variations on the growth conditions and target composition of sputtered cuprous telluride thin films, as reported by various authors^5,7–9^, may give the advantage of tuning the electrical and optical properties. In addition, there are not thorough reports on the vibrational properties of the system. Consequently, Raman and infrared (IR) spectra remain unknown for all the Cu_2−x_Te phases. The current lack of information on this technologically relevant material is due to the complexity introduced not only by its polymorphism, but also because it is a copper-vacancy-doped material. Herein, a systematic study of the structural, vibrational and electrical properties of Cu_2−x_Te thin films grown at a substrate temperature of 200 °C is reported. A Raman study, including a comparison with density functional theory (DFT) calculations of the CuTe phase (vulcanite), is presented. For the growth of the films all the sputtering targets were fabricated by cold pressing elemental copper and tellurium powders, varying the copper to tellurium nominal concentration ratio [Cu]/[Te]. The Cu_7_Te_5_ crystalline phase was favoured when [Cu]/[Te] was 1.5 and 1.75. Raman active modes corresponding to single phase CuTe as well as bands arising from films with mixture of phases were observed. Scanning probe microscopy (SPM) is a useful technique with high space resolution for evaluating local properties of heterogeneous microcrystalline materials. Recently, local properties of solar cells and photovoltaic materials used in solar cells have been evaluated using SPM techniques^10,11^. Kelvin probe force microscopy (KPFM) is one of the key SPM techniques to evaluate electrostatic properties of materials at the nanoscale^12,13^. KPFM is a tool based on conventional atomic force microscopy (AFM) that measures the surface potential difference between the AFM tip and the sample surface, which is related with the work function of the material. An important feature of amplitude modulated KPFM is that it allows to produce maps of both topography and surface potential simultaneously with high spatial resolution (~25 nm) and sensitivity (a few millivolts)^14^. For the sake of improvement of photovoltaic materials, it is significant to understand local and microcrystalline properties of these materials. In this work, to further extend the characterization of Cu_2−x_Te thin films, the average surface potential of the films and nanoscale properties of crystal clusters were analyzed by KPFM.

Amplitude modulated KPFM, or AM-KPFM, is a dual-pass SPM technique in which during the first pass, the cantilever is mechanically vibrated near its resonant frequency by a small piezoelectric element and the topographic features of the sample are recorded (Tapping mode). During a second pass, the cantilever is elevated to a given constant scan height, the mechanical oscillation applied to the tip is turned off and an oscillating voltage *U*_*AC*_*sin*(*ωt*) with a direct current (*U*_*DC*_) offset is applied directly to the AFM tip.

1 $$U(t)={U}_{AC}\,\sin \,\omega t+{U}_{DC},$$

Then the cantilever follows the recorded surface topography at a specific lift height above the sample while responding to electric influences on second pass (interleave) scan (trace and retrace). When there is a DC voltage difference between the tip and sample (contact potential difference), an oscillating electric force results acting on the AFM tip at the frequency ω (*F*_*ω*_). Since KPFM is a nulling technique, the ω component is used as feedback signal to adjust *U*_*DC*_ so that *F*_*ω*_ = 0,

2 $${F}_{\omega }=\frac{\partial C}{\partial h}{U}_{ac}({U}_{DC}-{{\rm{\Phi }}}_{s})\cos \,\omega t,$$

Where *C* is the capacitance in air formed by the surface of the film and the tip, and *h* is the distance between them. In experimental conditions, the AFM tip has its own surface potential (*Φ*_*t*_) depending on the material. *U*_*DC*_ corresponds to the local contact potential difference (*Φ*_*s*_):

3 $${U}_{DC}={{\rm{\Phi }}}_{t}-{{\rm{\Phi }}}_{s}.$$

If the tip and sample are at the same DC voltage, there is no force on the cantilever at frequency ω and the cantilever amplitude will be equal to zero. The voltage applied to the probe tip is recorded by the controller to construct a voltage map of the surface. In which brighter regions correspond to higher surface potential values, and darker regions to smaller surface potential values.

## Results and Discussion

### Structural properties

**Figure 1:**
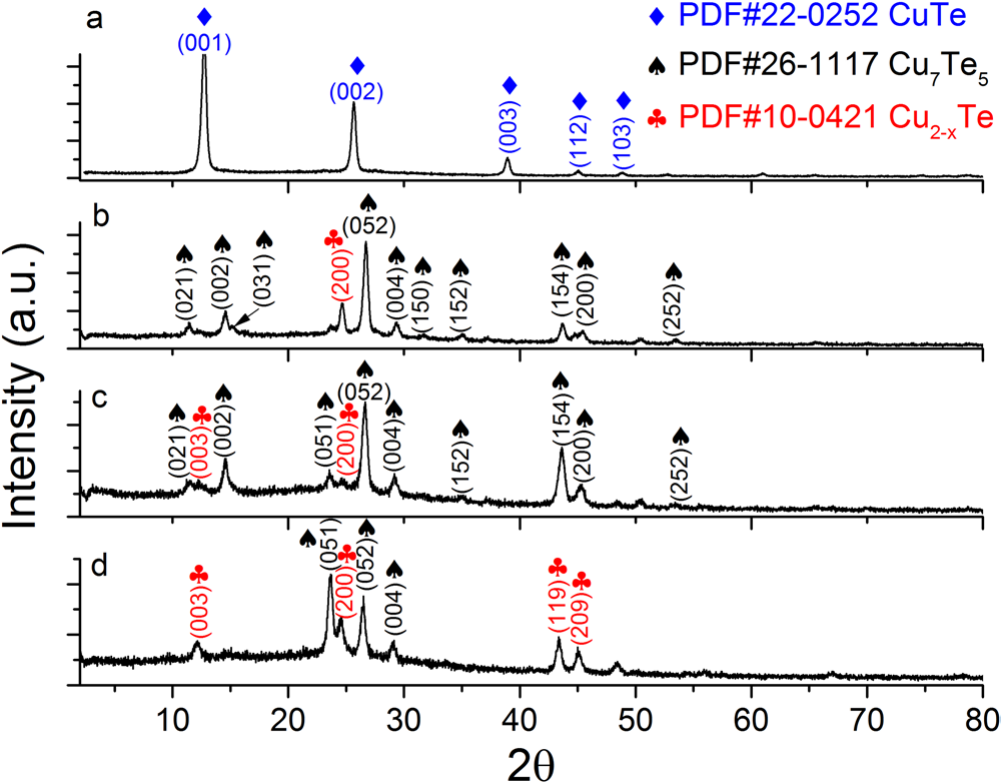
Diffraction patterns of the sputtered films with increasing [Cu]/[Te] ratio. From (**a**) to (**d**) 1.25, 1.5, 1.75 and 2, respectively. The obtained phases were vulcanite (CuTe), rickardite (Cu_7_Te_5_) and weissite (Cu_2−x_Te).

XRD measurements were performed on the films to study the structural changes upon the systematic addition of Cu to the targets. Figure 1a shows the diffractogram of the thin film grown using the target with [Cu]/[Te] = 1.25. The first three principal diffraction planes located between 10 and 40 degrees formed with preferential direction parallel to (0 0 *c*). These planes belong to the crystalline phase of CuTe (PDF #22–0252), often referred to as vulcanite. There are other low intensity peaks located at higher angles which also correspond to the same crystalline phase. This is a remarkable result, since most of Cu_2−x_Te thin films reported to date show significant mixtures of crystalline phases, whereas this thin film crystalline structure includes *only* the vulcanite phase. Moreover, this phase was not observed in any of the other films grown from the other targets.

The rest of the thin films fabricated comprised a mixture of phases. The diffractogram of the sample fabricated out of the [Cu]/[Te] = 1.5 target is shown in Fig. 1b. Here, the crystalline phase Cu_7_Te_5_ is dominant, whose highest intensity peak corresponds to the plane (052) located at 26.66 degrees; whereas there is little contribution of the hexagonal weissite phase PDF#10–0421 (space group P3m1, No. 156) with the diffraction plane (200) located at 24.73 degrees. It is worth clarifying that in the PDF#10–0421 this copper telluride phase (Weissite) is labelled generically as Cu_2−x_Te; thus, we have followed the same notation in Fig. 1 and throughout this work. Similarly, the diffraction pattern of the film sputtered using a target with concentration ratio [Cu]/[Te] of 1.75 presents the same high-intensity peaks, corresponding to the same mixture of phases: rickardite and weissite, Fig. 1c. For [Cu]/[Te] = 2, Fig. 1d, the dominant role of Cu_7_Te_5_ observed in the previous two cases is reduced due to an increased presence of the hexagonal weissite phase Cu_2−x_Te, through the appearance of diffraction lines corresponding to planes (119) and (209) at angles higher than 40 degrees.

The Cu-Te phase diagram is one of the most complex of the copper-chalcogen family^15^. This is a factor that may produce that small variations in the [Cu]/[Te] ratio result in the growth of different phases. Regarding a possible reproducibility issue on this complex system, it can be mentioned that our group recently reported^16^ prior work where films were deposited varying not only the [Cu]/[Te] ratio, but also the substrate temperature from RT to 350 °C. It was observed that T_s_ = 200 °C was an interesting temperature for copper telluride deposition, since nearly pure phases of CuTe and Cu_7_Te_5_ could be obtained and also the resistivity values were the lowest, compared to those grown at other substrate temperatures. The phases formed for the various [Cu]/[Te] ratios in Fig. 1 were similar to those reported previously for the same substrate temperature^16^. That is, it was found the same characteristic phase mixture of Cu_7_Te_5_ and Cu_2−x_Te for higher values of Cu in the targets, nearly pure Cu_7_Te_5_ for the middle ones, and the formation of the pure phase CuTe (vulcanite) for the lowest [Cu]/[Te] ratios (1.25 in this work and 1.0 in ref.^16^). In short, phase reproducibility is good, with small variations that do not affect the characteristic dominant phase for each [Cu]/[Te] ratio.

### Vibrational properties

#### Density functional theory results

**Figure 2:**
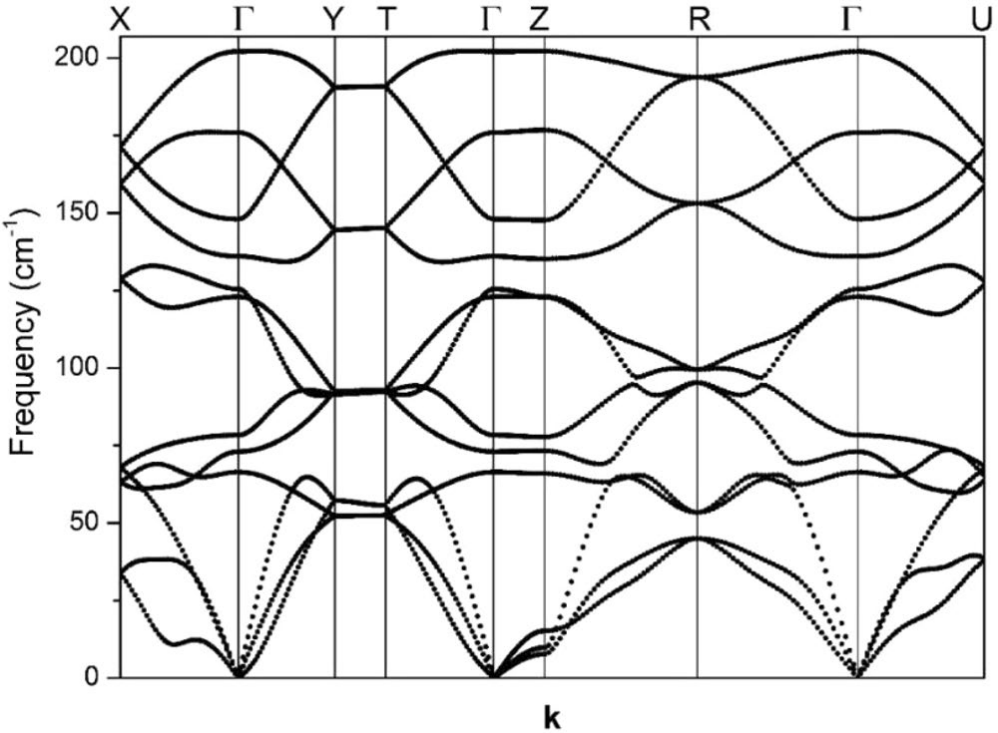
Phonon dispersion curves of CuTe (vulcanite).

**Figure 3:**
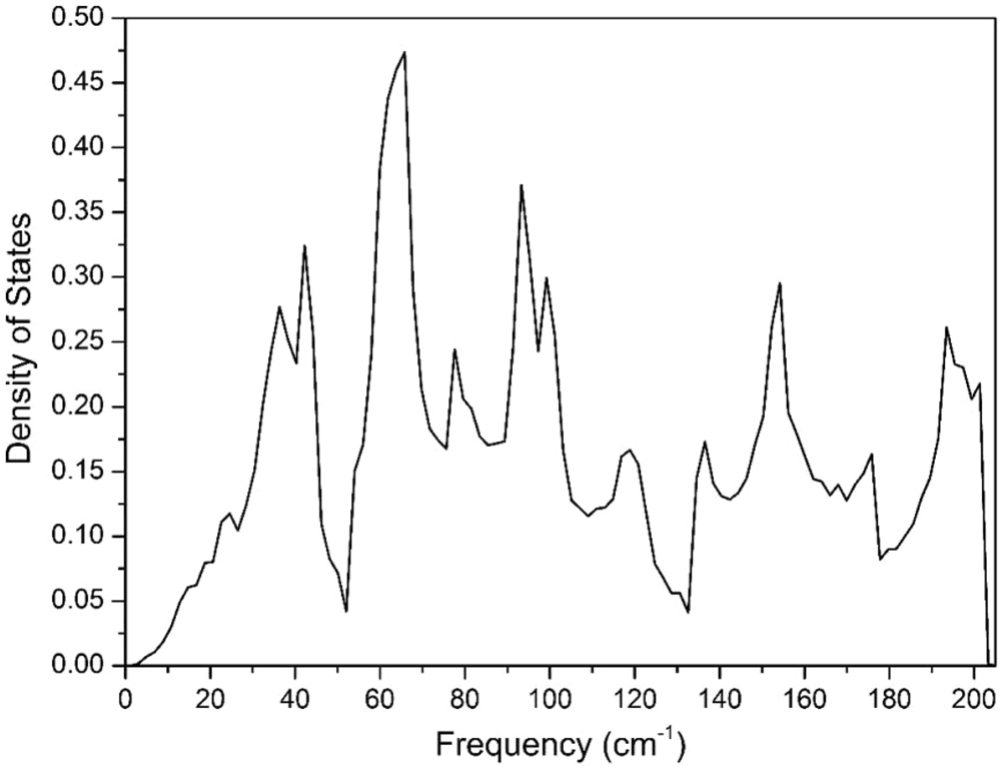
Phonon density of states of CuTe (vulcanite).

As mentioned above, reports on the vibrational properties of the various phases of the copper telluride Cu_2−x_Te system are scarce and limited. Among all the phases of the copper-telluride system, the 1:1 CuTe vulcanite phase (symmetry space group 59, *Pmmn*) has the simplest crystalline structure. This phase with orthorhombic symmetry has 4 atoms per unit cell. This characteristic makes it a suitable candidate for its study through first principles DFT calculations. Especially important is the fact that this structure does not contain randomly occupied copper vacancies, as opposed to most of the copper telluride phases. In our case, since it was possible to obtain single-phase vulcanite films, *i.e*. with no phase mixtures, the experimental measurements could be compared to the theoretical DFT predictions. Even though the calculations were performed only for one of the copper telluride phases, its understanding provides important insights about the vibrational properties of the whole system. Lattice parameters and atomic positions were optimized through minimizing the forces acting on each atom. The lattice parameters of the relaxed structure were *a* = 3.26, *b* = 4.02 and *c* = 7.58 Å, while the reported values stand as *a* = 3.16, *b* = 4.08 and *c* = 6.93 Å, with all the unit cell angles remaining at 90°. This represents a change in the lattice parameters of 3.16%, 1.47% and 9.38%, respectively. The differences between the experimental and calculated values for *a* and *b* are not significant considering the involved structural relaxation process; however, the difference in *c* is larger. This can be accounted for if one considers that vulcanite has a layered structure, in which layers are held together by Van der Waals-type forces. DFT calculations are less accurate when this kind of weak interactions are present because of their time-dependent nature. In this sense there are some corrections to deal with this problem, although they are not always effective. In this work it was chosen not to introduce any corrections in the calculations since for our purposes, *i.e*. calculation of the vibrational frequencies of a layered material, the vibrational frequencies (and most of the physical properties) do not depend strongly on the *c*-parameter due to the weak nature of the interaction perpendicular to the *a-b* planes. Binding energy and heat of formation per formula-unit were calculated to be E_(CuTe)_ = −6.13 eV/formula unit (or −590.64 KJ/mol) and ∆H_f_ = −0.14 eV (or −13.49 KJ/mol). Once the structures were relaxed, the phonon dispersion curves (Fig. 2) and vibrational density of states (Fig. 3) were computed using the software PHONON and VASP as external *ab initio* DFT software. The irreducible representations of the vibrational optical modes of CuTe (vulcanite) at the Brillouin zone center are:

$${\Gamma }_{opt}=2{A}_{g}+2{B}_{2g}+2{B}_{3g}+{B}_{1u}+{B}_{2u}+{B}_{3u}.$$

**Figure 4:**
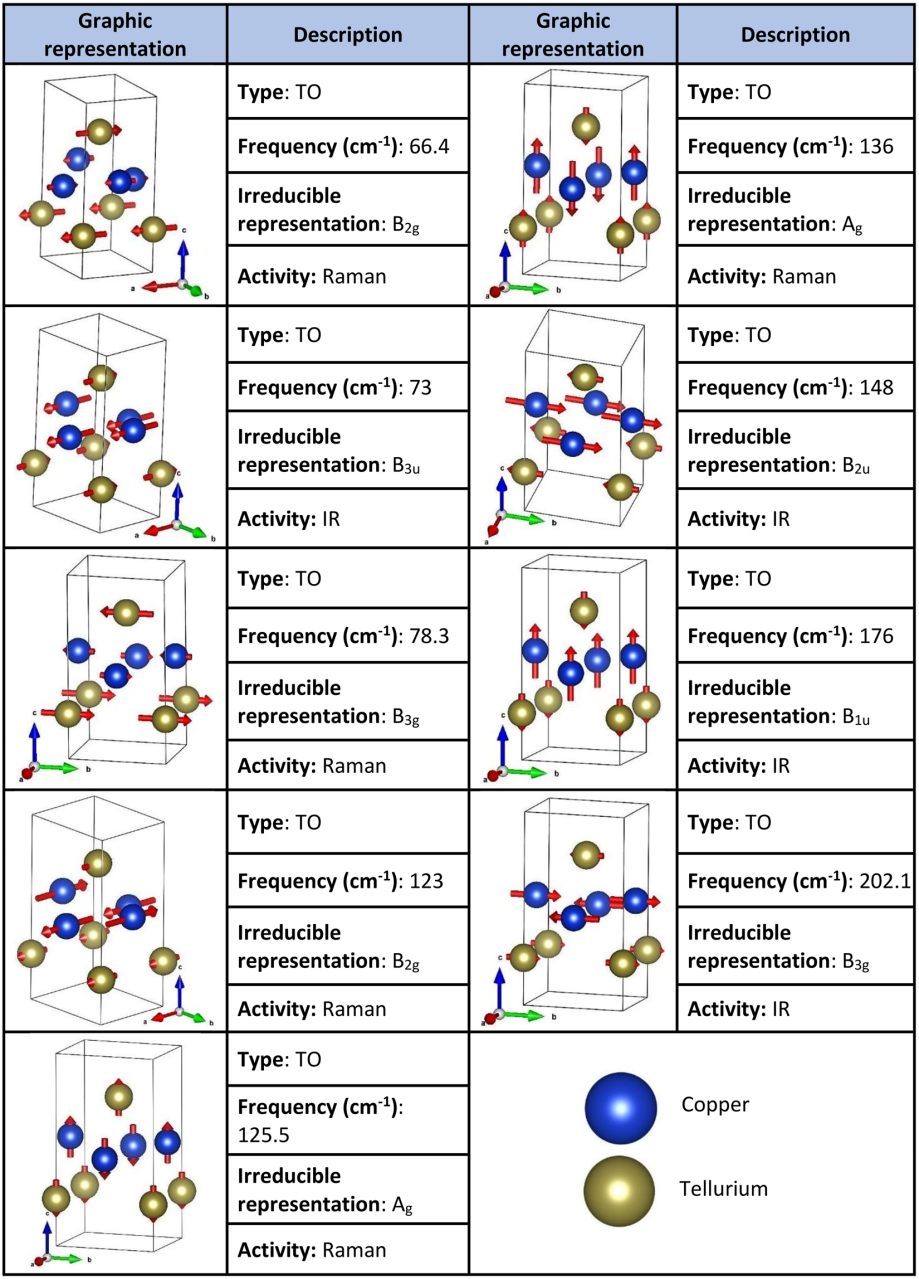
Raman and IR active modes of vulcanite (CuTe). The atomic motion in the unit cell for each normal mode is described. Other data include: type (transverse optical or longitudinal optical), frequency and irreducible representation for each vibrational mode.

Six of them (2A_g_ + 2B_2g_ + 2B_3g_) are Raman active, while the rest have IR activity. In Fig. 4 the atomic displacements, type, activity, frequency and irreducible representation of each optical mode are provided. All the images in this table were made using VESTA^17^.

### Raman Spectra

**Figure 5:**
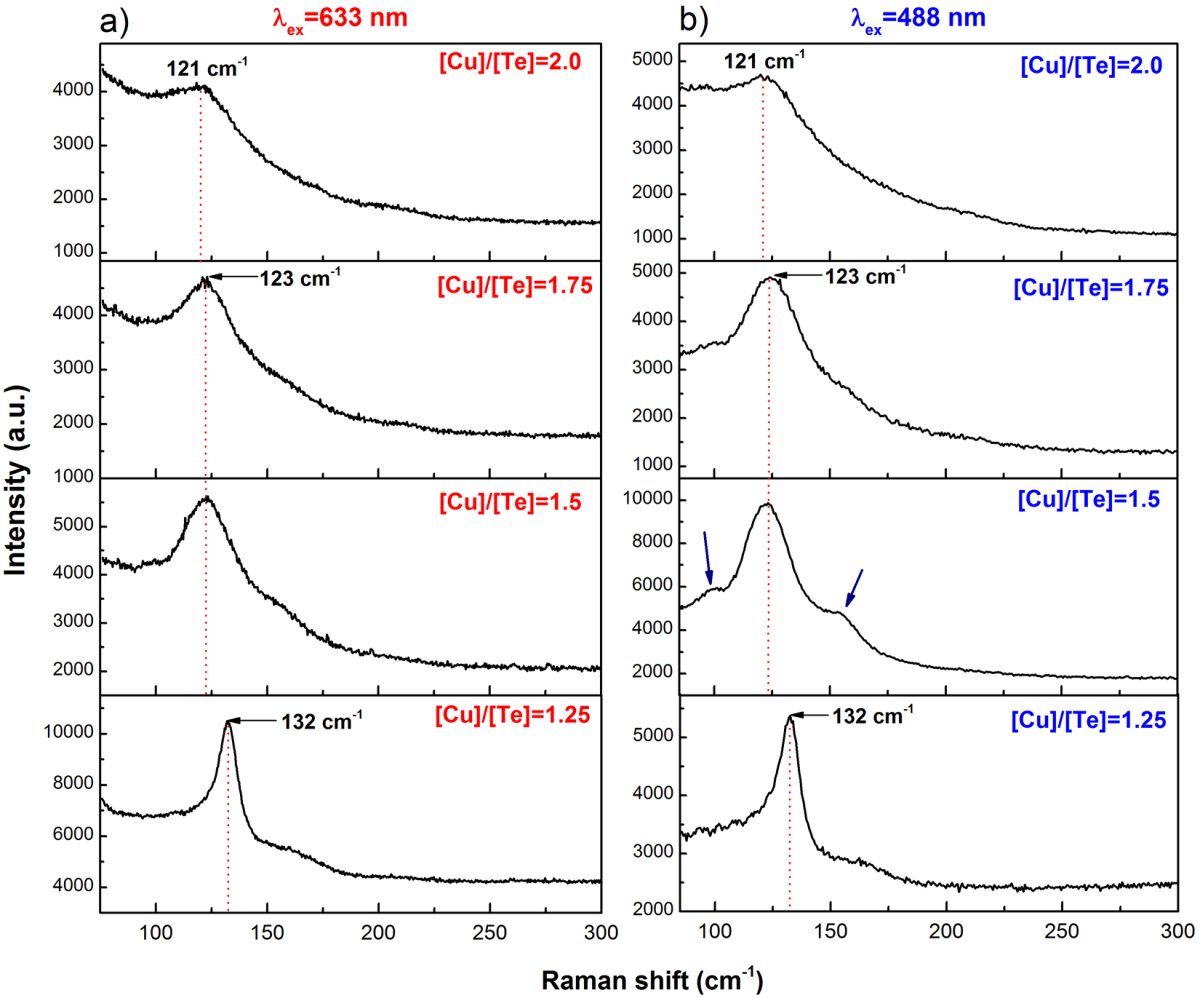
(**a**) Raman spectra of the copper telluride films obtained for the laser excitation line of λ_ex_ = 633 nm. (**b**) Raman spectra of the thin films measured with λ_ex_ = 488 nm. Besides the main peak, in the spectrum of the film grown from the [Cu]/[Te] = 1.5 two vibrational modes (indicated by the arrows) have intensities larger than the monotonically decaying signal (MDS) observable in all spectra. These two modes at the sides of the main peak have higher relative intensity than in the corresponding spectrum in Fig. 5a. This is an indication that resonance conditions (i.e. band-to-band transitions) are better matched when the excitation line λ_ex_ = 488 nm (2.54 eV) is used.

Raman spectroscopy is a suitable alternative to obtain experimental data of the vibrational frequencies at the Brillouin zone center of crystalline materials. As mentioned above, there are not previous reports of Raman spectra for any of the phases of the Cu_2−x_Te system. Here we compare the calculated DFT vibrational frequencies with the experimental data obtained from our measurements. Raman spectra were acquired for all the films obtained with the different [Cu]/[Te] ratios. Two different excitation laser wavelengths (λ_ex_) were used: 633 and 488 nm. All the measurements for each wavelength were carried out with the same power and acquisition time, that is 180 s for λ_ex_ = 633 nm (Fig. 5a) and 120 s for λ_ex_ = 488 nm (Fig. 5b). Two excitation lines were employed in order to verify that all the features in the spectra, namely the peaks and the accompanying monotonically decaying background, correspond to inelastic light scattering, as opposed to other kind of excitations such as photoluminescence.

The spectra corresponding to the film made with the [Cu]/[Te] = 1.25 target, whose crystalline phase is only vulcanite (CuTe), show one strong peak at 132 cm^−1^ for both excitation lines. This frequency is close to the value of 136 cm^−1^ calculated for one of the A_g_ modes of CuTe. Totally symmetric A_g_ modes usually present the strongest intensity of the vibrational modes because of the large change in susceptibility (and consequently of polarizability) induced by the complete out-of-phase motion of the oscillating ions. It is thus reasonable to identify the mode at 132 cm^−1^ as the A_g_ mode. For the Raman modes calculated at 123 (B_2g_) and 125.5 cm^−1^ (A_g_), these may be overlapped at the low-frequency side of the main peak, which indeed shows the corresponding asymmetry (bottom spectra, Fig. 5a,b). The spectra of the samples grown from targets with ratios [Cu]/[Te] = 1.5 and 1.75 show an intense peak at 123 cm^−1^, while the one grown from the target with [Cu]/[Te] = 2 has a maximum at 121 cm^−1^. In analogy to the case of vulcanite, it is plausible that these high-intensity peaks correspond to totally symmetric modes of the phases present on each film. It is to be noticed that the frequency of the most-intense peaks in Fig. 5 have a clear dependence on their structural characteristics, that is, on the dominant copper telluride phases for each sample, Fig. 1. The frequency of the most intense peak for vulcanite is 132 cm^−1^ (CuTe phase), the frequency of the strongest peak for samples with [Cu]/[Te] = 1.5 and 1.75 is 123 cm^−1^ (Cu_7_Te_5_ is the dominant phase for both samples), and the frequency of the main broad peak for sample [Cu]/[Te] = 2 (mixture of Cu_7_Te_5_ and Cu_2−x_Te) is 121 cm^−1^. Indeed, considering that the vibrational normal modes depend on the symmetry properties of the crystalline structure, it is expected that the frequencies of the modes present some variations from one polytype to another since they correspond to different vibrational normal modes.

**Figure 6:**
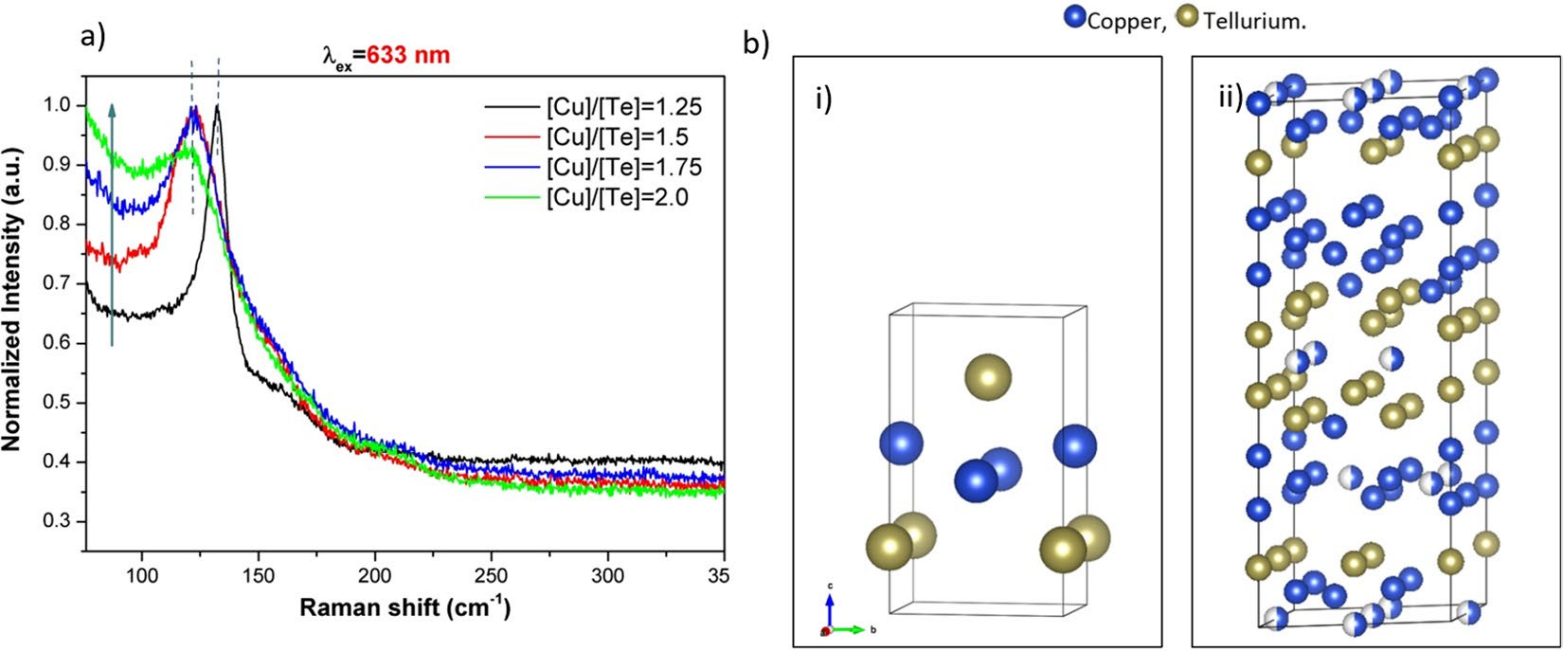
(**a**) Normalized Raman spectra obtained with the He-Ne laser line. The spectra have not been displaced vertically so that the increment of the monotonically decaying signal (MDS) at low wavenumbers increases as the value of [Cu]/[Te] rises, as indicated by the arrow; (**b**) comparison of the crystalline structure of (i) vulcanite (CuTe) and (ii) weissite (Cu_2−x_Te). The former does not include copper vacancies, while the latter possesses copper lattice sites randomly occupied. These are indicated by partially filled spheres, with the filling being proportional to the occupation probability.

Before referring to the calculated modes of vulcanite at 78 and 66 cm^−1^, we comment on the monotonically decaying signal (MDS) observed in all the spectra in Fig. 5. Figure 6a shows the normalized (to the highest intensity recorded for each spectrum) Raman spectra for λ_ex_ = 633 nm. Besides the shift of the main peak and its broadening for the higher [Cu]/[Te] concentrations, it is clear that the relative intensity of the MDS increases as the [Cu]/[Te] ratio gets larger, as indicated by the arrow. It is expected that other Raman active modes are immersed within such signal, such as the calculated modes at 78.3 and 66.4 cm^−1^ in the case of vulcanite. We note, however, that the latter was out of our measurement range (>75 cm^−1^). In the case of the films with phase mixtures, the MDS must have contributions of a rather large number of Raman active modes. Indeed, the number of atoms in the primitive cell (N) of Cu_7_Te_5_ is 48 and approximately 72 in the case of weissite Cu_2−x_Te^18^. Since the number of optical vibrational modes varies as 3N-3^19^, the number of Raman active modes will increase accordingly (the rule 3N-3 includes Raman, infrared as well as silent modes). That is, a large number of Raman bands must be convoluted within the MDS; however, it is quite unlikely that the intensities of those bands are from high-to-low when the frequency of the vibrational modes increases, so as to yield the observed shape of the MDS. It is reasonable then to hypothesize that the observed MDS has additional contributions that make it prevail over the intensity of most of the vibrational bands. Possible Rayleigh scattering from rough surfaces cannot not explain the observed behavior of the MDS. The RMS roughness obtained from AFM measurements for samples with [Cu]/[Te] = 1.25, 1.5, 1.75 and 2.0 were 9.4, 38.2, 12.9 and 11.9 nm, respectively. As it may be noticed, there is no correlation between the values of the RMS roughness and the intensity of the MDS, Fig. 6a. To gain insight into the origin of this inelastically scattered light, it must be recalled that copper tellurides are *p*-type materials with rather large free hole densities of the order of 10^20^–10^21^ cm^−3^^2,7^. For the films reported here, the free carrier density was of the order of 10^21^ cm^−3^ (in two cases only slightly above 10^22^ cm^−3^), *vide infra*. For such large free carrier densities, inelastic light scattering can be expected since electronic density fluctuations can couple to phonons^20^. Indeed, plasmon-phonon coupled modes have been observed at room temperature in *p*-type semiconductors such as GaSb^21^ and GaAs^22^, whose intensity increases as the doping level increased. In other words, the intensity of phonon-plasmon coupled modes scales with the free carrier density. In the case presented here, however, the intensity of the MDS does not scale fully as the hole density data (see below). That is, the free hole density increases and then slowly decreases as the [Cu]/[Te] ratio gets larger. This is not quite the behavior of the MDS in Fig. 6a, which systematically increases as the value of [Cu]/[Te] does. That is, besides the coupled electronic (hole) scattering, another contribution to the observed MDS can be inferred. At this point it is worth recalling that the Raman scattering cross section depends on the electric susceptibility changes generated by the oscillating ions during the normal modes vibrations. X-ray diffraction results showed a trend in the crystalline phases present in the films. As the [Cu]/[Te] rises, the presence of the weissite phase (Cu_2−x_Te) increases, Fig. 1. This is relevant because, unlike the vulcanite phase (Fig. 6b,i), both the Cu_7_Te_5_ and Cu_2−x_Te structures present some Cu sites with probability of occupation less than one. This is illustrated in the case of weissite in Fig. 6b.ii with the partially filled Cu sites. Such vacancy sites disrupt the symmetry of the underlying crystalline structure yielding significant local-field contributions to the electric susceptibility^23^. The X-ray diffraction results, thus, indicate that the higher the value of [Cu]/[Te], the larger the number of Cu sites not fully (randomly) occupied, yielding larger susceptibility changes as the local-field contributions become more significant. This, in turn, enhances the observation of inelastic scattering of light by the charged vacancies in the material. That is, vacancies become highly effective scattering centers as the oscillating ions around vacancies induce local-field variations. In summary, it is hypothesized that the MDS is the result of three contributions: (i) convoluted vibrational modes, with the most intense above the level of the MDS; (ii) plasmon-phonon coupled modes; and (iii) copper-vacancy related local-field contributions to the electric susceptibility. The Raman spectra of vulcanite (bottom spectra, Fig. 5a,b) would have only the first two contributions since randomly occupied sites do not occur in its structure.

**Figure 7:**
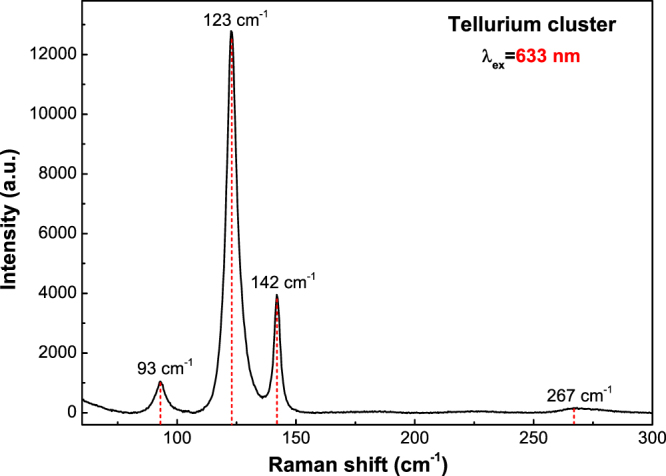
Raman spectra of a photo-modified spot corresponding to a tellurium cluster obtained with λ_ex_ = 633 nm.

Another important issue about the measurement of the Raman spectra of the system Cu_2−x_Te is that the compounds of this system have a certain degree of photo-sensibility. In this sense, all measurements had to be done cautiously under low laser power in order to avoid modifying the samples. It was observed that the Raman spectra of laser-damaged regions correspond to tellurium^24–27^, which has a very intense Raman signal with its main peak at 123 cm^−1^ (Fig. 6). Three reasons can be mentioned to associate to copper tellurides the peaks with frequencies close to 123 cm^−1^ in the Raman spectra in Fig. 5 (as opposed to being the result of photo-degradation). First, after making a measurement in a photo-modified spot, the affected zone becomes notably darker, as observed through the spectrometer microscope. Second, if tellurium clusters were present in the films, the sharp Te peak at 142 cm^−1^ (Fig. 7) should be observed, which does not become apparent, nonetheless, in any of the spectra in Fig. 5. Finally, one can mention that the intensities of the Raman active modes of the Cu_2−x_Te system are substantially lower than those of tellurium. The spectrum shown in Fig. 7 was obtained with the same power and laser wavelength as the spectra of Fig. 5a, but with an acquisition time six times smaller. However, the S/N ratio of the Te spectrum in Fig. 7 is significantly higher than those in Fig. 5a. With these observations it is effortless to note whether a sample has been damaged or not during its exposure to the laser beam. From all these considerations it possible to be certain that the Raman spectra presented in Fig. 5 correspond to unmodified regions of the films and, therefore, to copper telluride phases.

Infrared spectroscopy yields as well important information on the lattice dynamics of materials. Attenuated total reflection measurements were carried out in the 100–1800 cm^−1^ range for the whole set of films. However, the high carrier density prevented obtaining any useful feature to obtain the infrared active vibrational modes of any of the films. Only a monotonically increasing signal was obtained.

### Electrical properties

**Figure 8:**
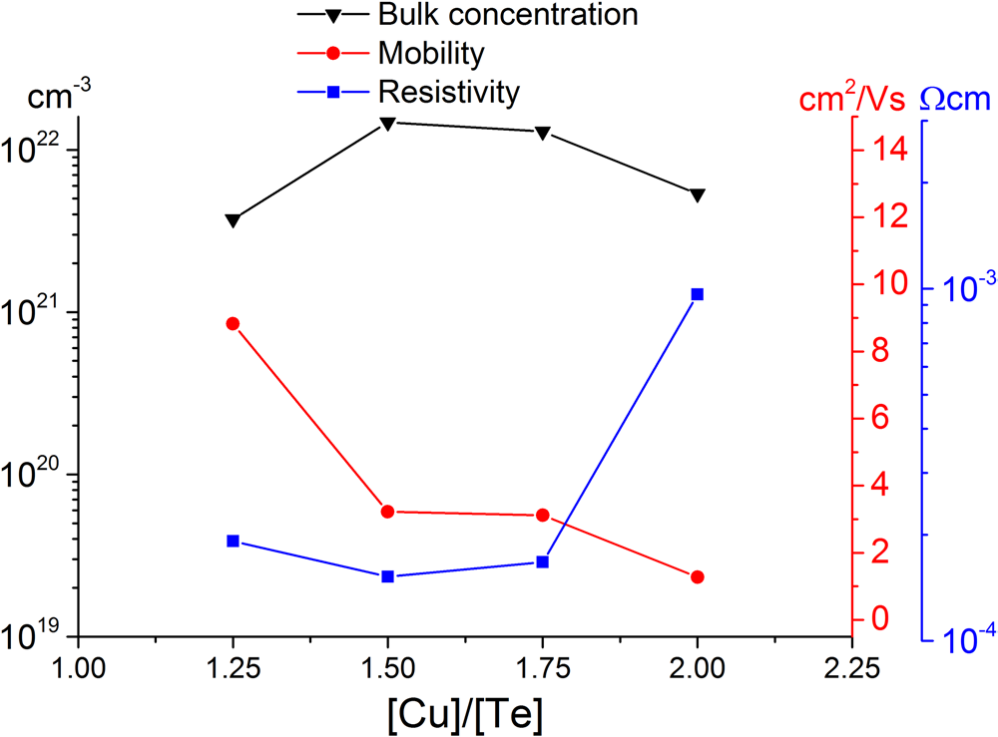
Experimental bulk carrier concentration, mobility and resistivity of the sputtered films, as a function of target nominal composition [Cu]/[Te].

Previous studies report that the Cu_2−x_Te system is a *p*-type material with typically hole concentrations between 10^20^–10^21^ cm^−3^, and resistivities of the order of 10^−4^ Ω cm^2,7^. In this work, the most conductive sample had a resistivity of 1.52 × 10^−4^ Ω cm, with a mobility of 3.22 cm^2^/Vs and carrier density of 1.48 × 10^22^ cm^−3^. Figure 8 shows the resistivity, mobility and carrier concentration for all films. The thicknesses of the samples, measured by AFM, are summarized in Table 1. To determine the average thickness of the films, the scratch method was utilized. A sharp scratch was produced on the sample using a steel scalpel. Subsequently, standard AFM tapping mode was performed to measure the step height of the scratch. At least five different areas were measured and averaged for each sample to yield the corresponding standard deviation. It may be noticed that the films grown from the targets [Cu]/[Te] = 1.5 and 1.75 show comparable electrical properties, in correspondence with the fact that both samples have similar crystalline structure (*i.e*. copper telluride phases), as shown in Fig. 1.

**Table 1:** Thicknesses of the films deposited from the target with the indicated compositions.

Sample [Cu]/[Te]	thickness (nm)
1.25	421 ± 21
1.5	458 ± 58
1.75	530 ± 47
2	308 ± 23

### Kelvin probe surface microscopy

**Figure 9:**
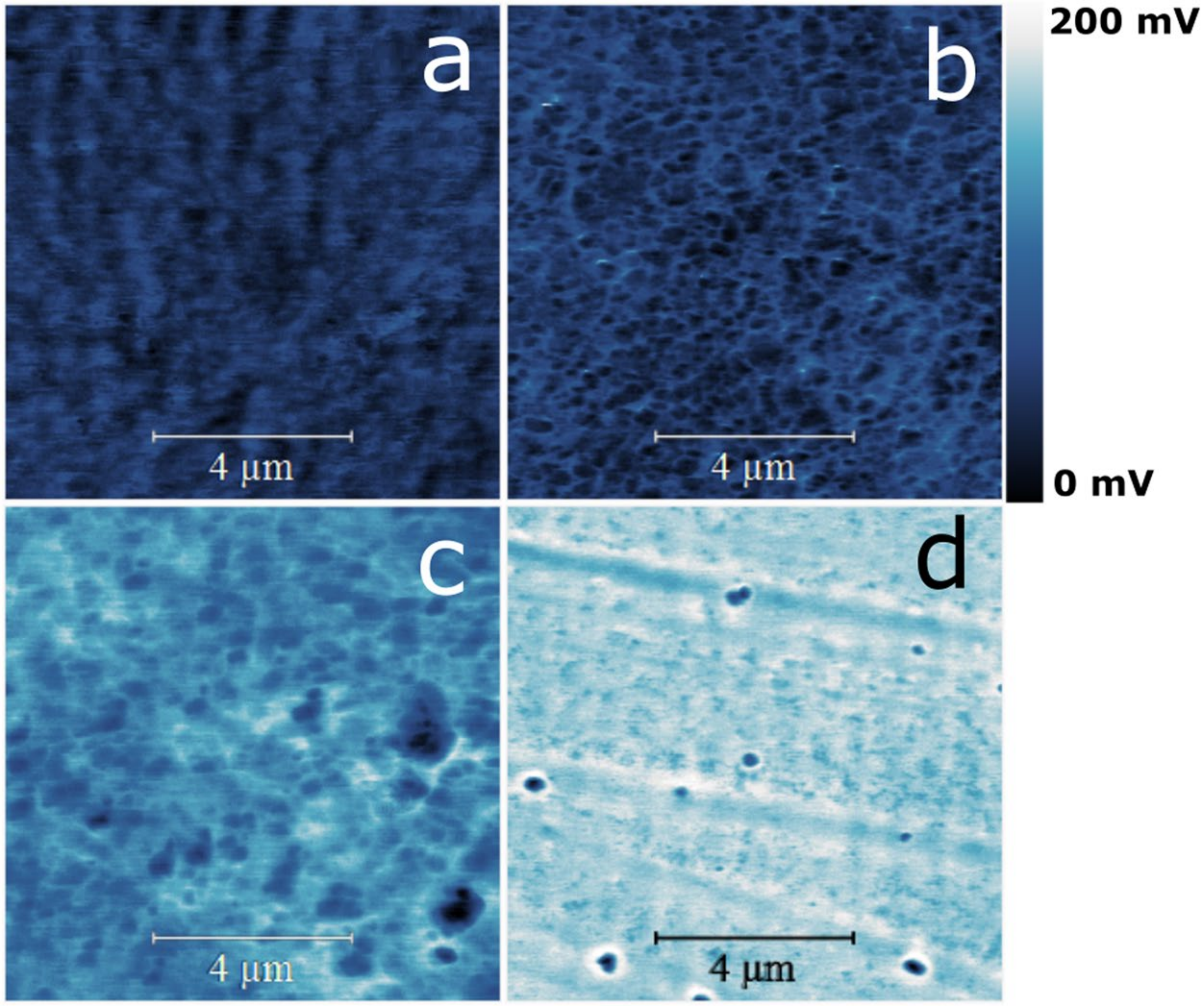
Kelvin probe force microscopy (KPFM) surface potential maps (10 × 10 μm^2^) of samples (**a**) [Cu]/[Te] = 1.25, (**b**) [Cu]/[Te] = 1.5, (**c**) [Cu]/[Te] = 1.75 and (**d**) [Cu]/[Te] = 2.

**Figure 10:**
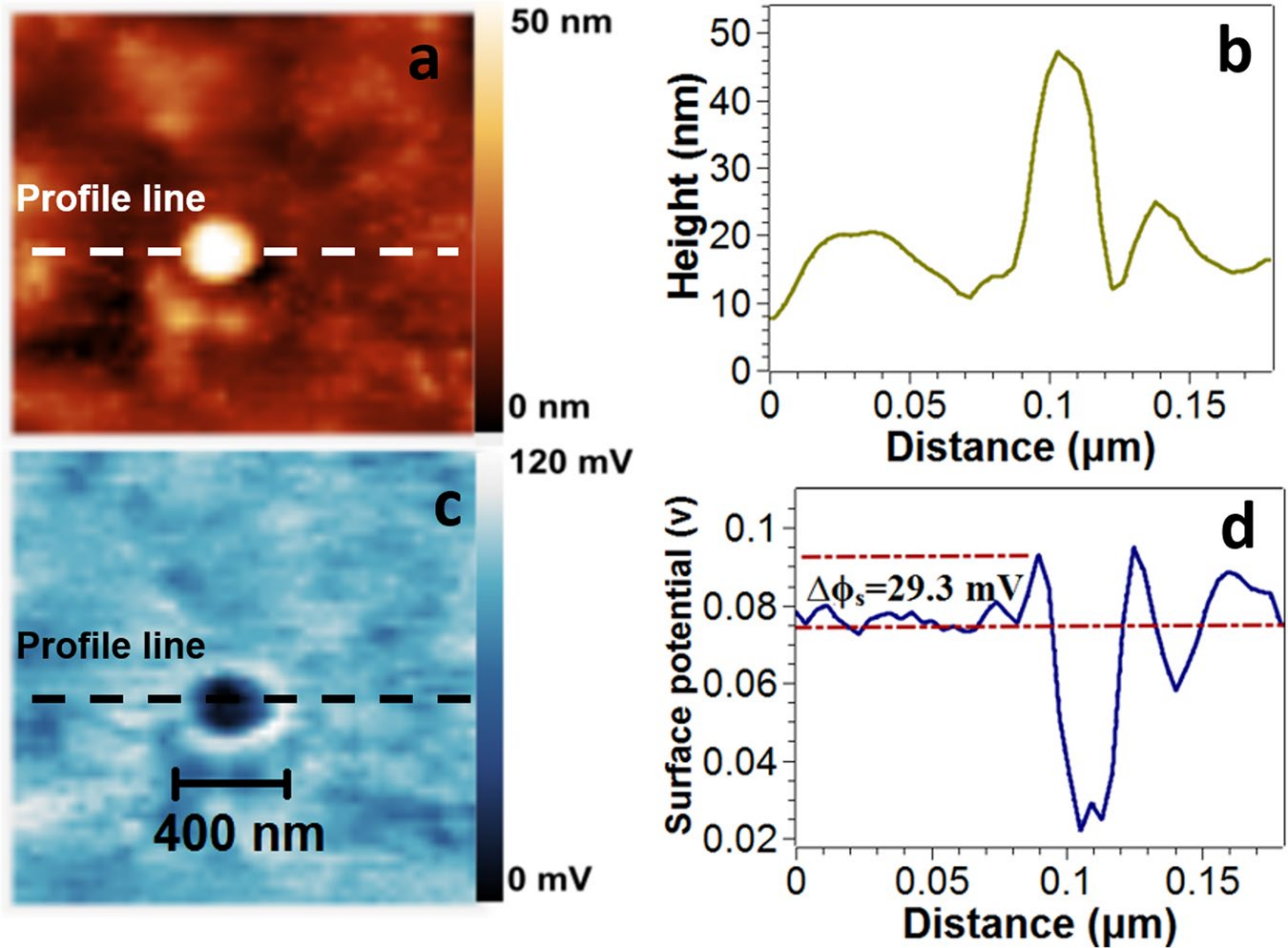
Representative images of the [Cu]/[Te] = 2.0 film obtained by AFM and KPFM of a crystal cluster. (**a**) Topographic image obtained on tapping mode. (**b**) Height profile of the indicated line. (**c**) Surface potential distribution. (**d**) Surface potential profile along the indicated line.

Micrographs of the surface potential distribution of all the samples are shown in Fig. 9. Brighter areas in the surface morphology and surface potential images correspond to larger surface height and surface potential, respectively. All films display homogenous surface structure with similar surface potential roughness. Figure 9d, *i.e*. sample [Cu]/[Te] = 2, confirms the formation of well-defined crystalline clusters. In Fig. 10, a detailed image of the grains of the sample [Cu]/[Te] = 2 is provided. An increment on the surface potential of around 29.3 mV above the mean surface potential value is observed at the grain boundaries, effect which has been reported formerly in thin films^28,29^. Previous studies have demonstrated that the formation of such clusters on polycrystalline semiconductor materials strongly contributes to creating localized states at the grain boundaries, thus producing separation centers at these locations. Consequently, majority charge carriers migrate to the intra-grain regions, whereas minority charge carriers get trapped at the grain boundaries^28–31^.

**Figure 11:**
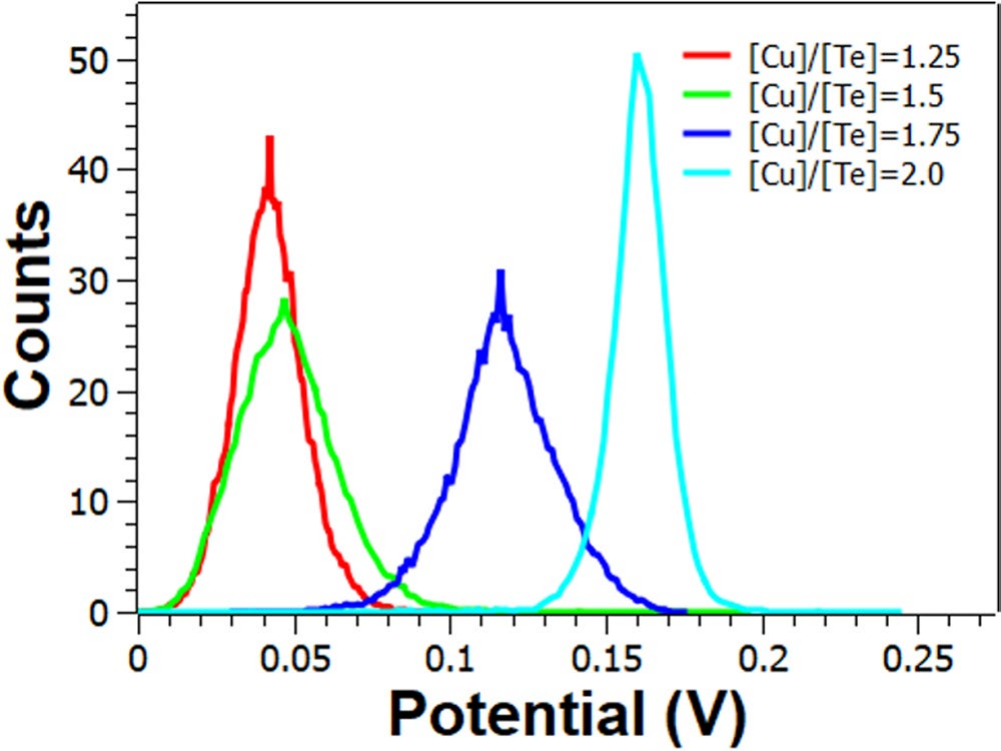
Surface potential distribution from the 10 × 10 μm^2^ scan area of the surface potential mapping images.

Figure 11 shows the surface potential distributions over 10 × 10 μm^2^ scan areas for all the [Cu]/[Te] ratios. As observed, the surface potential distributions differ from each other. All distributions present a constant shift to the positive surface potential direction depending upon the [Cu]/[Te] ratio. Mean values of samples [Cu]/[Te] = 1.25, [Cu]/[Te] = 1.5, [Cu]/[Te] = 1.75 and [Cu]/[Te] = 2.0 were found to be 42, 48, 116 and 160 mV, respectively. That is, the higher the [Cu]/[Te] ratio, the larger the overall surface potential. The surface potential at the air-surface interface has a bulk and a surface component. The former, arises from the electronic density in the solid. The latter, or surface dipole moment, derives from a redistribution of charges at the surface due to the loss of periodicity. It is reasonable to assume that these two contributions are affected by the charge centers produced by copper vacancies present in the structure. This is in agreement with the fact that the surface potential increases with the [Cu]/[Te] ratio, Figs 9 and 11, and with the increment in copper-vacancy density for the copper-rich polytypes. Figure 6b shows the crystalline structure of CuTe and Cu_2−x_Te, where the existence of copper vacancies in the copper-rich phase is noticeable.

## Conclusions

It has been shown that Cu_2−x_Te films can be prepared by sputtering using cold-pressed single targets fabricated from Te and Cu powders in appropriate [Cu]/[Te] ratios. X-ray diffraction showed that when [Cu]/[Te] = 1.25, vulcanite (CuTe) was the only copper telluride phase obtained. Further addition of Cu into the targets yields the growth of a mixture of phases: rickardite (Cu_7_Te_5_) and weissite (Cu_2−x_Te). Experimental Raman spectra, and calculated phonon dispersion curves and one-phonon density of states for vulcanite, obtained from Density Functional Theory, are reported for the first time. Good agreement between theoretical and experimental results was found, although it was not possible to identify all the predicted Raman modes. In addition, the Raman spectra of films with mixture of phases for the Cu_2−x_Te system were successfully obtained. Understanding of the vibrational behaviour of vulcanite, the simplest structure of the copper telluride family, helped to gain insight about the vibrational properties of the more complex phases. The Raman spectra of the films were superimposed onto low-frequency monotonically decaying signals, whose intensity was proportional to the [Cu]/[Te] ratio. These MDSs have been ascribed to contributions of (i) convoluted vibrational modes, (ii) phonon-plasmon coupled modes, and (iii) copper-vacancy related local-field variations. In the particular case of vulcanite, only the first two contributions may occur since its structure lacks copper sites partially occupied. Surface potential maps of all samples were successfully obtained by KPFM, which showed that the films shifted to higher surface potential as the amount of Cu was increased. Similarly, a detailed map of the structure of the topographic structure and surface potential at the nanoscale was obtained. These measurements indicated a substantial increase of the local surface potential at the boundaries of the crystal grains. Furthermore, KPFM images suggests that at the highest value of Cu concentration (*i.e*. [Cu]/[Te] = 2), formation of larger crystal clusters was promoted. The overall surface potential was proportional to the value of the [Cu]/[Te] ratio.

## Methods

In this work, all the targets used to sputter the films were prepared by cold pressing elemental copper and tellurium powders. The powders consisted of copper powder 3-µm average particle size, 99.7% trace metals basis, and tellurium powder, ~30 mesh and 99.7% purity. For simplicity, herein the targets are referred to as [Cu]/[Te] = X, where X represents the Cu-to-Te ratio of the target. Four targets with the following nominal composition [Cu]/[Te] were fabricated: 1.25, 1.5, 1.75 and 2. Each target was pressed in air for 30 minutes at a pressure of 16 tons in a stainless steel mould. The Argon working pressure was kept at 5 mTorr for all growths. The target-substrate distance was 5 cm, with a deposition time of one hour at a substrate temperature (T_s_) of 200 °C. Cleaned rectangular Corning glass slides (2.5 cm × 7.5 cm) were used as substrates for all depositions and the radio frequency power source operated at 20 W. The plasma was ignited under an ultra-high purity Ar gas flux. Prior to each growth, the target surface was cleaned by the plasma, with a pre-erosion time of 5 minutes. X-ray diffraction (XRD) experiments were carried out in diffractometer equipped with a copper target, operating at 30 kV and 20 mA, with an incidence angle of 2.5 degrees. The sweeping angle 2θ ranged from 2 to 80 degrees. All the patterns were identified using the Powder Diffraction Files (PDF) provided by the International Centre for Diffraction Data (ICDD) database and the software MDI Jade 6. Raman studies were performed with excitation wavelengths of 633 and 488 nm in a state-of-the-art high-resolution micro-Raman spectrometer. The micro Raman spectra were obtained using a 100 × microscope objective (i.e. backscattering configuration) to focus the laser beam on the samples surface. For this microscope objective the probed area was a spot of ~1 micron in diameter for the employed excitation wavelengths. Electrical properties measurements were made in a Hall effect system by the Van der Pauw method at room temperature (20 °C). For this purpose, small graphite contacts were deposited on the corners of 1 cm^2^ samples. Also, film thickness and surface potential mapping by KPFM were performed in an atomic force microscope using an uncoated Antimony (n-type) doped Si tip, with an oscillation frequency of 340.5 Hz. For estimating the film thickness, taping mode was used at 0.5 Hz (tip velocity = 0.996 μm/sec) scan rate and a resolution of 256 lines on different areas at the edge of the film. Furthermore, a step analysis was also carried out. To generate surface potential maps, dual-pass KPFM was performed by recording simultaneously topography in tapping mode (1^st^ pass) and surface potential in lift mode (2^nd^ pass). For this purpose, the same tip was used and its lock-in phase was found to be −180° by the fine tuning feedback method previously reported^32^. This was verified by the electrical tuning function in the Dimension Icon (Veeco) AFM software. In the interleave mode, a drive amplitude of 5000 mV and a lift height of 15 nm were selected. Surface potential distributions were obtained by selecting areas of 10 µm by 10 µm on each sample. Analyses of both topographic and potential images were carried out using Gwyddion - SPM data analysis software, with no further post-processing beyond mean plane subtraction leveling. Scanning probe experiments were performed in air at room temperature (∼25 °C).

### Computational details

Density functional theory (DFT) calculations were made using the VASP code^33–36^ with the help of Perdew-Burke-Ernzerhof (PBE) pseudopotentials of the generalized gradient approximation (GGA)^37,38^. The cut off energy for the plane waves was set to 550 eV, while the energy convergence parameter for two consecutive self-consistent steps was fixed at 10^−6^ eV. Relaxation of lattice parameters as well as atomic positions were carried out until the Hellman-Feynman forces were less than 0.01 eV/Å. In the Monkhorst–Pack scheme a 19 × 15 × 9 mesh was used to relax the structure. For the calculation of vibrational properties, the software PHONON^39,40^ along with VASP as complementary DFT software were utilized. For vibrational properties purposes a 3 × 3 × 2 supercell (72 atoms) and a 6 × 4 × 3 mesh were employed. For this calculation, the accuracy in energy for two consecutive self-consistent steps was raised to 10^−8^ eV.

### Data Availability

The datasets generated during the current study are available from the corresponding author on reasonable request.
